# Highly Heterogeneous Morphology of Cobalt Oxide Nanostructures for the Development of Sensitive and Selective Ascorbic Acid Non-Enzymatic Sensor

**DOI:** 10.3390/bios13010147

**Published:** 2023-01-16

**Authors:** Abdul Sattar Chang, Aneela Tahira, Fouzia Chang, Abdul Ghaffar Solangi, Muhammad Ali Bhatti, Brigitte Vigolo, Ayman Nafady, Zafar Hussain Ibupoto

**Affiliations:** 1 Dr. M. A. Kazi Institute of Chemistry, University of Sindh Jamshoro, Jamshoro 76080, Sindh, Pakistan; 2 Institute of Chemistry, Shah Abdul Latif University of Khairpur Mirs, Khairpur Mirs 66111, Sindh, Pakistan; 3 National Center of Excellent in Analytical Chemistry, University of Sindh Jamshoro, Jamshoro 76080, Sindh, Pakistan; 4 Institute of Environmental Sciences, University of Sindh Jamshoro, Jamshoro 76080, Sindh, Pakistan; 5 Institut Jean Lamour (CNRS, IJL), Université de Lorraine, F-54000 Nancy, France; 6 Department of Chemistry, College of Science, King Saud University, Riyadh 11451, Saudi Arabia

**Keywords:** Co_3_O_4_ nanostructures, sodium citrate, non-enzymatic approach, ascorbic acid sensor

## Abstract

The surface tailored metal oxide nanostructures for the development of non-enzymatic sensors are highly demanded, but it is a big task due to the wide range of complexities during the growth process. The presented study focused on the surface modification of the heterogeneous morphology of cobalt oxide (Co_3_O_4_) prepared by the hydrothermal method. Further surface modification was conducted with the use of sodium citrate as a reducing and surface modifying agent for the Co_3_O_4_ nanostructures through the high density of oxygenated terminal groups from the citrate ions. The citrate ions enabled a significant surface modification of the Co_3_O_4_ nanostructures, which further improved the electrochemical properties of the Co_3_O_4_ material toward the design of the non-enzymatic ascorbic acid sensor in a phosphate buffer solution of pH 7.4. The morphology and crystal arrays of the Co_3_O_4_ nanostructures were studied by scanning electron microscopy (SEM) and powder X-ray diffraction (XRD) techniques. These physical characterizations showed the highly tailored surface features of Co_3_O_4_ nanostructures and a significant impact on the crystal properties. The electrochemical activity of Co_3_O_4_ was studied by chronoamperometry, linear sweep voltammetry, and cyclic voltammetry (CV) for the detection of ascorbic acid. The linear range of the proposed sensor was measured from 0.5 mM to 6.5 mM and a low limit of detection of 0.001 mM was also estimated. The presented Co_3_O_4_ nanostructures exhibited significant surface roughness and surface area, consequently playing a vital role toward the selective, sensitive, and stable detection of ascorbic acid. The use of a low cost surface modifying agent such as sodium citrate could be of great interest for the surface roughness and high surface area of nanostructured materials for the improved electrochemical properties for the biomedical, energy storage, and conversion systems.

## 1. Introduction

The physiological activities in living organisms are intensively governed by the presence of ascorbic acid (vitamin C), and an abnormal level of ascorbic acid concentration can severely affect health and cause scurvy, followed by the formation of collagen. Ascorbic acid is highly useful in our body for the development of the skin, immune, and tissue system [1]. Importantly, ascorbic acid is a strong antioxidant and it drives the metabolism of cholesterol effectively in the human body [2], hence its accurate and sensitive determination is vital for practical applications. Conventional methods have been used to determine the ascorbic acid from food and clinical samples [3,4], and these methods include chromatographic [5] spectrophotometry [6], fluorimetry [7], NIR, MIR, and FT-Raman techniques [8]. These conventional methods are associated with high cost, time taking, complicated, and require well skilled people for the detection of ascorbic acid. Furthermore, electroanalytical methods such as voltammetry [9,10,11,12], amperometry [13], and potentiometric [14] have been used extensively due to their high potential, promising, and advantageous features such as low cost, simple, highly sensitive, and accuracy toward the detection of analytes [15]. Electrochemical methods have been used through two methodologies for the development of biosensors. First, the use of enzyme immobilization on electrocatalytic materials [16], dye immobilization [17], and polymer coatings [18] for the determination of ascorbic acid. Enzyme immobilization on electrocatalytic materials suffers from poor thermal stability, enzyme spoilage, a complicated enzyme immobilization process, and a high consumption of substrate molecules [19]. Second, recently, non-enzymatic methods have been intensively used for the design of electrochemical sensors due to their several advantages such as being highly sensitive, thermally stable, easy to operate, and fast response [20]. However, non-enzymatic methods require efficient electrocatalytic materials for the sensitive and selective detection of small biomolecules, hence this method is still at an early stage with regard to its utilization in practical applications. Thus, new studies are always welcome by researchers and industrialists for the synthesis of new functional materials with enhanced electrocatalytic properties for sensitive and selective electroanalytical applications. Among the materials, transition metal oxides are a unique class of materials due to their unique d-orbital configuration for the tailored catalytic properties for the development of non-enzymatic sensors. Cobalt oxide (Co_3_O_4_), among the transition oxides, has been highly investigated for its electrochemical applications for two decades due to its spinel structure and mixed oxidation states for swift redox reaction kinetics. Moreover, it is low cost, ecofriendly, and easy to prepare [21]. This is the reason why Co_3_O_4_ nanostructures have been studied for a wide range of applications such as energy storage systems [22], heterogeneous catalysis [23], magneto resistive devices [24], and electrochromic thin films [25]. Aside from these applications of Co_3_O_4_ nanostructures, they have been potentially utilized for the development of electrochemical sensors [26]. For example, Co_3_O_4_ nanostructures have been employed for the development of glucose [27,28], lactic acid [29], ascorbic acid [30], and urea [31] electrochemical sensors. The improvement in the electrocatalytic properties of Co_3_O_4_ nanostructures was carried out by various methods such as doping, developing composite structures, and surface modifications using different preparation methods [19,20,31,32]. Despite these extensive efforts, the electrochemical performance of Co_3_O_4_ nanostructures has been found to be poor toward the fabrication of efficient non-enzymatic electrochemical sensors and their realization in real sample analysis. Therefore, new types of simple and low cost approaches for the synthesis of Co_3_O_4_ nanostructures have to be studied for the development of a new generation of non-enzymatic ascorbic acid sensors. The use of oxygen rich oxygen terminated atoms of anionic species such as citrate can be effectively used to evolve the morphology and change the number of surface active sites on the surface of the material for the enhanced electrochemical properties of Co_3_O_4_ nanostructures. The use of rich oxygenated terminal groups for enabling the surface roughness of Co_3_O_4_ nanostructures has not been studied elsewhere. For this reason, we employed sodium citrate due to its low cost reducing agent properties via oxygenated terminals for the Co_3_O_4_ nanostructures. Furthermore, in this study, we highlighted the role of citrate ions from sodium citrate with abundant oxygen terminals to create the roughness on the surface of the Co_3_O_4_ nanostructures. Additionally, the presented study was carried out in different ways to compare to our previous studies, where we directly utilized the surface modifying agents in the precursors of the nanostructured material [31,32]. In these studies, we did not highlight the surface roughness factor by the use of low cost and mild reducing agents for the prepared nanostructured materials toward the development of non-enzymatic sensors. Hence, the proposed research work was completely different from our previous studies and this kind of strategy has not been reported to date. In the end, the modified surface of the Co_3_O_4_ nanostructures played an important role in the sensitive and selective detection of ascorbic acid using a non-enzymatic approach.

## 2. Materials and Methods

### 2.1. Used Chemicals

Different chemicals such as cobalt sulfate, sodium citrate, urea, ascorbic acid, and 5% Nafion were purchased from Sigma Aldrich Karachi, Pakistan, and all of them were of high analytical grade. The preparation of the desired solutions was conducted with deionized water. Prior to the experiments, the glassware was cleaned with deionized water and dried at room temperature. The phosphate buffer solution of pH 7.4 was made with the use of various salts such as 0.1 mM H_3_PO_4_, 0.1 mMNaH_2_PO_4_, 0.1 mM Na_2_HPO_4_, and 0.01 mM NaCl in the deionized water. The pH of the phosphate buffer solution was adjusted using 0.2 M HCl and NaOH aqueous solutions.

### 2.2. Hydrothermal Preparation of Co_3_O_4_ Nanostructures Using Sodium Citrate as a Surface Modifying Agent

**Figure 1:**
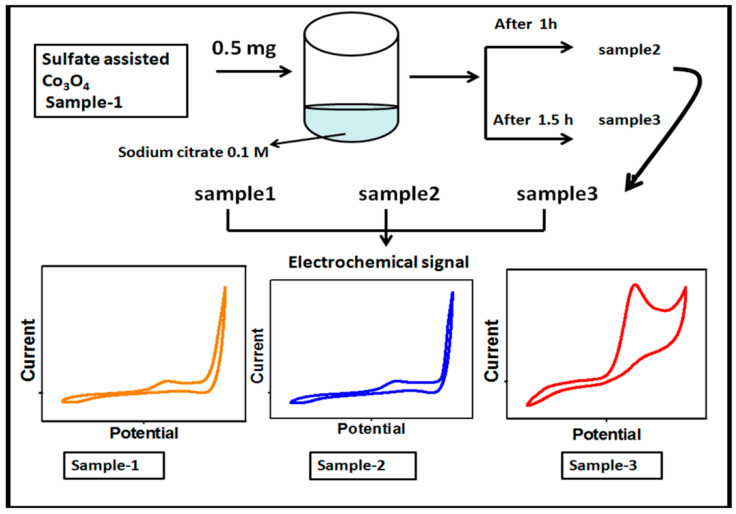
Schematic diagram for the preparation of S1, S2, and S3 samples of the Co_3_O_4_ nanostructures and their electrochemical characterization.

First, the Co_3_O_4_ nanostructures were prepared by the hydrothermal method followed by thermal annealing in air. The synthesis of the Co_3_O_4_ nanostructures was carried out with the use of a cobalt precursor of 0.1 M cobalt sulfate and 0.1 M urea in 500 mL of deionized water. The well dispersion of the cobalt precursor was obtained through mechanical stirring with a glass rod for 10 min. Then, the cobalt precursor solution was covered with an aluminum sheet and the hydrothermal process was performed at 90 °C to 95 °C for 5 h. The prepared cobalt hydroxide material was collected on ordinary filter paper and washed several times with deionized water followed by drying at 60 °C overnight. Then, the cobalt hydroxide phase material was kept in China clay made crucible and annealed at 500 °C for 4 h. Afterward, a typical Co_3_O_4_ nanostructure with a black color was obtained and named as pristine Co_3_O_4_ (sample 1). Second, the Co_3_O_4_ nanostructures were treated with sodium citrate for the purpose of surface modification. The choice of sodium citrate was taken on the basis of a rich source of oxygenated terminals that could easily tailor the surface roughness of the Co_3_O_4_ nanostructures. In the typical sodium citrate treatment, 5 g of the Co_3_O_4_ nanostructures were placed in a 100 beaker capacity with 20 mL of 0.1 M sodium citrate. The reducing agent treatment was carried out at two different times such as 1 and 1.5 h. After this, the modified Co_3_O_4_ nanostructures were collected on the filter paper, washed with deionized water, and dried overnight. The treated Co_3_O_4_ nanostructures for 1 and 1.5 h were labeled as sample 1 and sample 2, respectively. Each step involved during the synthesis of the surface modified Co_3_O_4_ nanostructures is shown in Figure 1.

### 2.3. Structural and Electrochemical Measurements for Ascorbic Detection on Citrate Derived Co_3_O_4_ Nanostructures

The crystal quality aspects of the Co_3_O_4_ nanostructures were studied by powder X-ray diffraction (PXRD) with measurement conditions of CuKα radiation (λ = 1.5418 Å) at 45 kV and 45 mA. A low resolution scanning electron microscopy was used under the experimental conditions of 20 kV to evaluate the surface morphology of the as prepared Co_3_O_4_ nanostructures. The Co_3_O_4_ nanostructure catalyst ink was prepared by dissolving 10 mg of the Co_3_O_4_ nanostructures into 2.5 mL of deionized water and 0.5 mL of 5% Nafion. Then, a homogenous catalyst ink was achieved in an ultrasonic bath for 10 min. For the cleaning of the glassy carbon electrode (GCE), it was polished with alumina paste (0.5 µm) and rubbed with silicon paper. Afterward, GCE was washed several times with the deionized water. Then, the drop cast method was used to deposit 10 µL of Co_3_O_4_ nanostructures onto the GCE, which was labeled as the modified (MGCE) and used as a working electrode. The electrochemical experiments were conducted with a three electrode cell configuration involving the silver–silver chloride (Ag/AgCl, 3.0 M KCl) as the reference electrode and platinum wire as a counter electrode. Keeping in mind the physiological pH environment for the real sample analysis of ascorbic acid in the human body, here, we used the phosphate buffer solution of pH 7.4. Furthermore, from the previous works, it has been shown that the electrochemical detection of ascorbic acid is more favorable around a pH of 7.3–7.4. Different ascorbic acid concentrations were made in a phosphate buffer solution of pH 7.4. The phosphate buffer solution of pH 7.4 was used as a supporting electrolyte. Different electrochemical modes were used such as cyclic voltammetry (CV), linear sweep voltammetry (LSV), and chronoamperometry. The real sample analysis was also conducted on the MGCE for the quantification of ascorbic acid from the human urine sample. The real sample was collected by a healthy voluntary person from our laboratory and the preparation was carried out by adding 1 mL of human urine sample in 19 mL of phosphate buffer solution at pH 7.4. This was then analyzed by the presented non-enzymatic ascorbic acid sensor.

## 3. Results and Discussion

### 3.1. Morphology, and Crystalline Characterization of Oxygenated Terminals Treated Co_3_O_4_ Nanostructures

**Figure 2:**
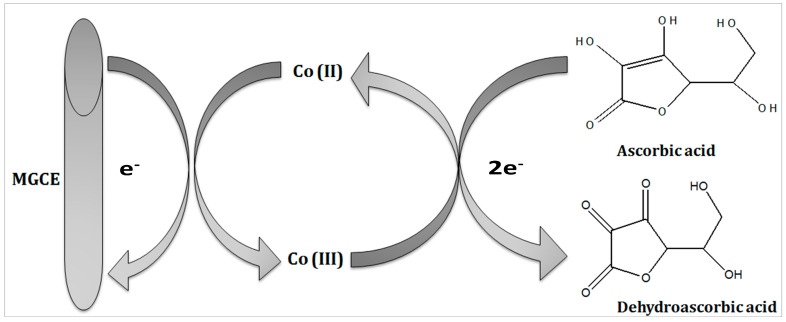
Illustration of the oxidation of ascorbic acid using the proposed Co_3_O_4_ nanostructures.

**Figure 3:**
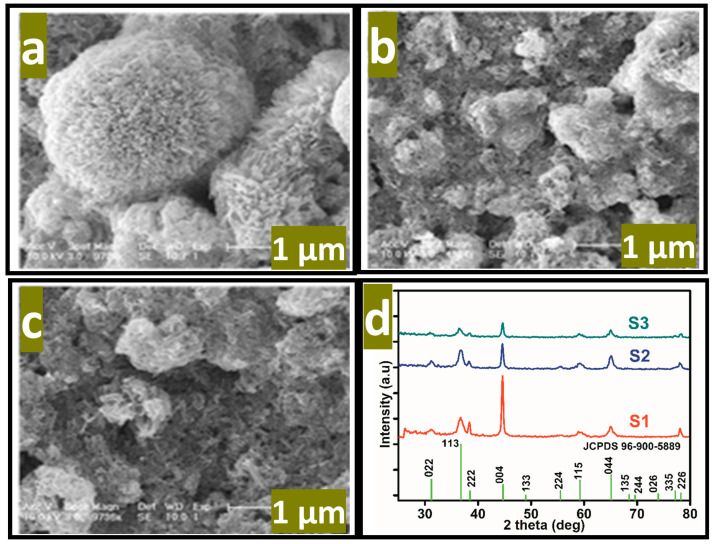
SEM images of the various Co_3_O_4_ nanostructures. (**a**) Sample-1, (**b**) sample-2, (**c**) sample-3, (**d**) the corresponding XRD patterns.

The reaction mechanism of ascorbic acid onto the Co_3_O_4_ nanostructure takes place by the transfer of two electrons and protons, as shown in Figure 2, and it has been generally represented in previous studies [32,33]. The Nernst equation has been used to describe the equal number of proton and electron transfer during the oxidation of ascorbic acid as given under:

Nernst equation, E_p_ (V) = −0.059 (m/n) pH + E^0^ (1)

where E_p_ is the peak potential, m and n are the number of protons and electrons, respectively. The ascorbic acid molecules were adsorbed on the modified GCE when it was inserted in the ascorbic acid solution, then the applied potential favored the oxidation of ascorbic acid into dehydroascorbic acid. At the same time, electrons and protons were produced, and consequently, the free electrons contributed toward the enhanced conductance of MGCE and also the electron transfer kinetics, as shown in Figure 2. During the electrochemical reaction of ascorbic acid on MGCE of the Co_3_O_4_ nanostructures, the formation of cobalt(II) hydroxide, and furthermore, its oxidation into CoOOH could be expected, hence such a phenomenon is considered to monitor the detection of ascorbic acid using a non-enzymatic approach. The morphology of the modified Co_3_O_4_ nanostructures was evaluated by SEM, as shown in Figure 3. The SEM image of the pristine Co_3_O_4_ nanostructures without sodium citrate (sample-1) exhibited a porous flower like structure with the dimensions of a few microns, as shown in Figure 3a. However, the citrate ion treated Co_3_O_4_ nanostructures with different time intervals at 1 and 1.5 h were also studied by SEM and their corresponding SEM images are shown in Figure 3b,c. The citrate ions made the surface of the Co_3_O_4_ nanostructures relatively rough due to its reducing aspects of sodium citrate, and these changes were highly visible for samples-2 and -3 through the SEM images, as shown in Figure 3b,c. The surface modification of the Co_3_O_4_ nanostructures after hydrothermal synthesis has not been investigated and the present approach offers a facile approach for the surface modification of nanostructured materials. The significant heterogeneity on the surface of Co_3_O_4_ could be seen in Figure 3b,c, which could be useful for electrochemical applications. These surface alterations were further proven to be an effective tool for the development of a non-enzymatic ascorbic acid sensor in the presented work. To investigate the purity and crystal quality of the as prepared Co_3_O_4_ materials, the powder XRD technique was performed as shown in Figure 3d. The experiment was performed at the 2 theta angle range with scanning from 30° to 80°. The XRD study showed that there was an effect on the intensity of the diffraction patterns when sodium citrate was used and some of the reflections of Co_3_O_4_ became more intense, suggesting that an improved crystal quality compared to the pristine Co_3_O_4._ The diffraction patterns of Co_3_O_4_ fully agreed with the standard JCPDS card (96-900-5889). The Co_3_O_4_ samples were identified with cubic crystal phase and there was no any other impurity in the samples, suggesting a high quality of the as prepared Co_3_O_4_ material.

### 3.2. Electrochemical Measurements for the Determination of Ascorbic Acid Using Surface Modified Co_3_O_4_ Nanostructures

**Figure 4:**
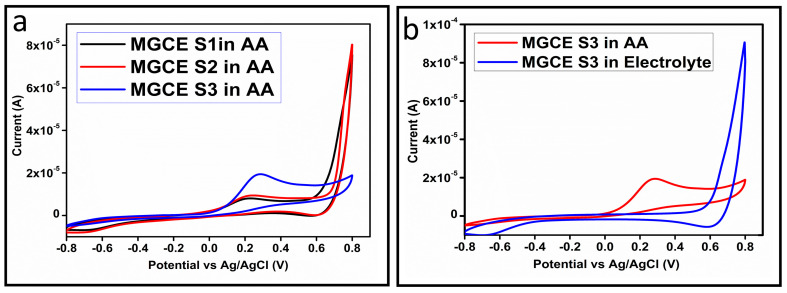
(**a**) Cyclic voltammogram of MGCE at a scan rate of 50 mV/s with the untreated S1, treated (S2 and S3) in 0.1 mM ascorbic acid. (**b**) Cyclic voltammogram of MGCE with sample-3 at a scan rate of 50 mV/s in 0.1 mM ascorbic acid and in the electrolyte.

**Figure 5:**
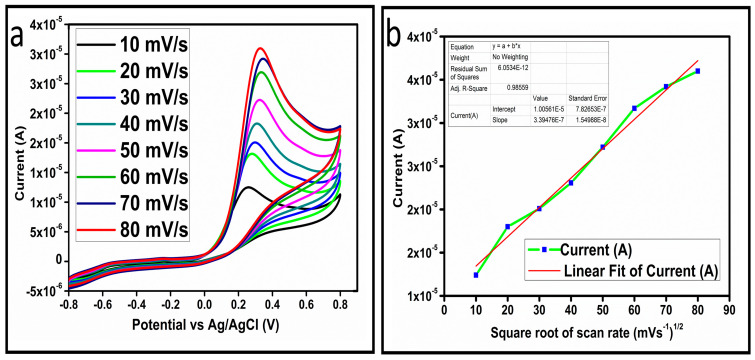
(**a**) Cyclic voltammogram of sample-3 (S3) with various scan rates in 0.1 mM ascorbic acid. (**b**) Plot of the oxidation of peak current versus square root of the scan rate.

**Figure 6:**
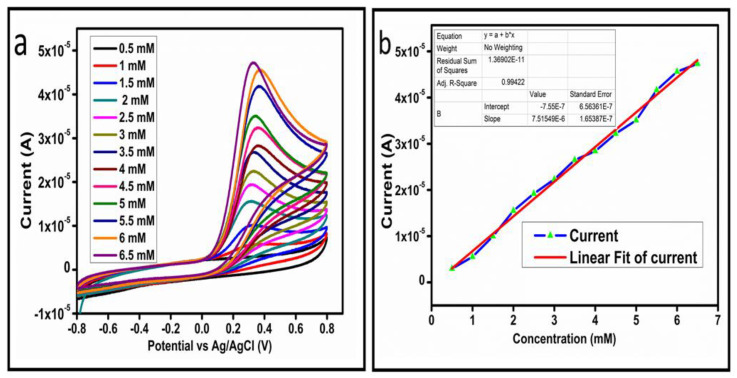
(**a**) Cyclic voltammograms at different concentrations of ascorbic acid at a scan rate of 50 mV/s. (**b**) Linear plot of the oxidation peak current versus the concentration.

**Figure 7:**
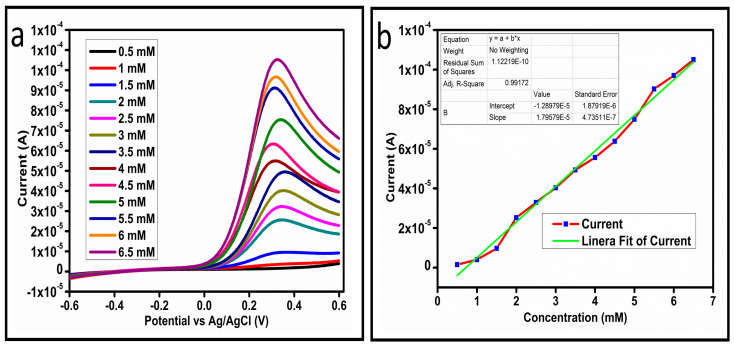
(**a**) LSV at different concentrations of ascorbic acid at 10 mV/s. (**b**) Linear fit of the current at different concentrations of ascorbic acid.

**Figure 8:**
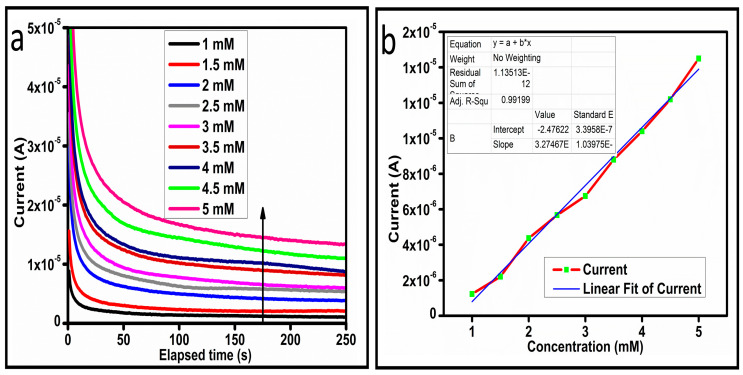
(**a**) Chronoamperometric signal at different concentrations of ascorbic acid against 0.3 V (Ag/AgCl). (**b**) Linear plot of the current versus concentration.

The CV curves at a scan rate of 50 mV/s were measured for the Co_3_O_4_ nanostructures in the absence and presence of ascorbic acid in a phosphate buffer solution of pH 7.4. The CV curves of the pristine Co_3_O_4_ nanostructures (sample-1) and surface modified Co_3_O_4_ nanostructures (sample-2, sample-3) deposited onto GCE were recorded in 0.1 mM ascorbic acid, as shown in Figure 4a. The electrocatalytic properties of three samples of the Co_3_O_4_ nanostructures were studied by CV curves and sample-3 was found with an enhanced oxidation peak current and well-shaped peak, as shown in Figure 4a. To further verify whether the peak came mainly from ascorbic acid or electrolyte, we only tested sample-3 in the electrolyte without ascorbic acid and with the use of ascorbic acid, as shown in Figure 4b. Sample-3 showed a non-Faradic process in the supporting electrolyte, however, it had a described well-resolved oxidation peak in the ascorbic acid, suggesting its superior redox electrochemical activity, as shown in Figure 4b. The surface area and excellent compatibility of the newly prepared Co_3_O_4_ nanostructures with the surface of GCE all played a vital role toward the superior performance during the non-enzymatic sensing of ascorbic acid in the presented study. Moreover, an enhanced surface roughness and improved catalytic properties of the Co_3_O_4_ nanostructures also played an outstanding role in the sensitive detection of ascorbic acid. Furthermore, we studied the Faradic kinetics of the Co_3_O_4_ nanostructures (sample-3) through CV curves at various scan rates ranging from 10 to 80 mV/s in 0.1 mM ascorbic acid, as shown in Figure 5a. It was observed that the oxidation peak current increased with each rise in the scan rate, suggesting the well-behaved diffusion electrochemical process on the modified electrode. The plot of the oxidation peak current enhancement with each scan rate was plotted against the square root of the scan root as shown in Figure 5b, indicating the good analytical features of the modified GCE [33,34]. The working range of the newly developed non-enzymatic ascorbic acid sensor based on Co_3_O_4_ nanostructures was also evaluated in a phosphate buffer solution of pH 7.4. Various CV curves measured at a scan rate of 50 mV/s in the different concentrations of ascorbic acid are shown in Figure 6a. It was obvious that the oxidation peaks increased linearly with each rise in increment in the ascorbic acid concentration, verifying the sensitive signal of sample-3 against the ascorbic acid molecules. The linear plot of the oxidation peak current of each CV curve was fitted against the ascorbic acid concentrations and well-defined analytical fitting features were observed through the regression coefficient of 0.99. This confirms the excellent analytical behavior of the presented electroanalytical method based on Co_3_O_4_ nanostructures, as shown in Figure 6b. A wide linear range of 0.1–6.5 mM ascorbic acid concentration was shown using CV measurements. The low limit of detection was estimated by published work [35], and it was found to be about 0.001 mM. The linear range and the low limit of detection of the presented ascorbic acid sensor were superior to many of the published ascorbic acid sensors using various nanostructured materials such as the Pt–Ti alloy [36,37,38]. In the CV curves, the Faradic current was directly used to quantify the kinetics of the electrochemical process occurring on the surface of the working electrode. Moreover, the peak current depends on the speed at which the electrode material receives a number of molecules from the bulk solution of analyte which are connected to mass transport. It has been shown that at a low concentration of analyte, diffusion takes place without disturbance, hence peak shift could not take place. However, diffusion is highly disrupted at higher concentrations, thus offers a big barrier for favorable mass transport, which is compensated by disruptive diffusion. Therefore, the reoccurrence of mass transport without the disturbance and electrochemical reaction needs more potential, thus it causes a shift in the peak potential. Furthermore, the electrochemical reaction needs a particular time span to assure the diffusion of reactive species and the charge transfer on the surface of the working electrode. Therefore, the shift in the potential could be attributed to the delay in the electrochemical process because of the shortness of the given time compared to the time given to the electrochemical reaction at a lower concentration or scan rate. The intersection of CV curves for the 6 mM and 6.5 mM concentrations could be indexed to the shift in the peak potential toward low overpotential for the 6.5 mM concentration, hence it seems that there is an intersection. The calibration of various ascorbic acid concentrations was also made with the use of linear sweep voltammetry (LSV), as shown in Figure 7a. It is clear that the CV results for calibration were further found to be in good agreement with the LSV results and offer another aspect of the sensitivity of the Co_3_O_4_ nanostructures. Interestingly, the LSV results became very sensitive due to their higher working range from 0.1 to 6.5 mM, as shown by the linear fitting of the oxidation peak current from the LSV curves versus the different ascorbic acid concentrations (Figure 7b). Furthermore, the calibration plot was obtained by using chronoamperometry, which is a more sensitive electrochemical method and the observed results are shown in Figure 8a. Each chronoamperometric response suggests the successive increase in the current related to a rise in the concentration of ascorbic acid, indicating the excellent electrocatalytic properties of the Co_3_O_4_ nanostructures tuned by the treatment of sodium citrate. We also plotted linear fitting through the increase in the current during the chronoamperometric signal against various ascorbic acid concentrations, as shown in Figure 8b. The linear fitting revealed the highly sensitive nature of the Co_3_O_4_ nanostructures by demonstrating a linear range of 1–5 mM of ascorbic acid. The chronoamperometry results suggest that each i–t curve response time is highly durable and can be applied for certain periods of time for the detection of ascorbic acid.

**Figure 9:**
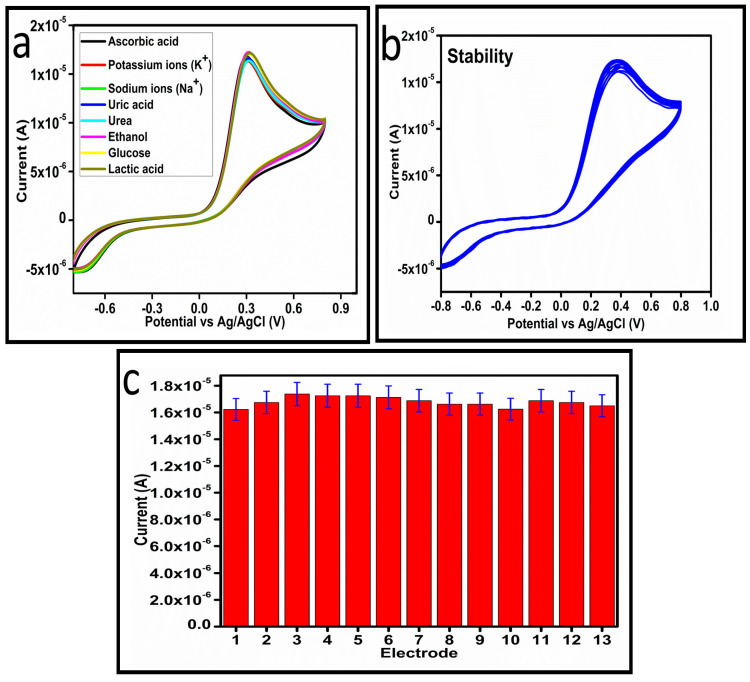
(**a**) Cyclic voltammogram of sample-3 in the presence and absence of different interfering substances at a scan rate of 50 mV/s. (**b**) Cyclic voltammogram of sample-3 for measuring the stability in 0.1 mM ascorbic acid at a scan rate of 50 mV/s. (**c**) A bar graph with error bars illustrates the consistency of CV analysis results.

The selectivity of the as prepared Co_3_O_4_ nanostructures toward ascorbic acid detection was studied under the environment of competing interfering agents such as uric acid, potassium ions, sodium ions, ethanol, lactic acid, urea, and glucose, as shown in Figure 9a. For the selectivity measurement, 0.1 mM ascorbic acid solution and other interfering agents were used and the standard addition method was used to record the change in the oxidation peak current during the recording of CV curves, as shown in Figure 8a. From this analysis, it is obvious that less than 3% change in the peak current was noticed with the addition of these interfering species, consequently, the prepared Co_3_O_4_ nanostructures showed a selective response toward the detection of ascorbic acid. Hence, the presented non-enzymatic ascorbic acid sensor has a high capability to quantify ascorbic acid from the complex matrices of real samples. The stability of the Co_3_O_4_ nanostructures was also evaluated in 0.1 mM ascorbic acid by measuring different CV curves at the scan rate of 50 mV/s, as shown in Figure 9b. The good stability could be attributed to the fact that sodium citrate added the significant surface roughness of the Co_3_O_4_ nanostructures, which firmly bonded with the surface of GCE, consequently resulting in the observation of excellent stability. For better analytical representation, the same stability results of the CV analysis were further described by a bar graph using an error bar, as shown in Figure 9c, suggesting an acceptable relative standard deviation (RSD) value of less than 1%. From 13 CV cycles of the same MGCE, this indicates the significant repeatable capability of the electrode during the stability measurement.

To study the practical aspects of the as developed non-enzymatic ascorbic acid sensor for the determination of ascorbic acid for human urine sample, we used the recovery (%) method as given in Table 1. The recoveries of the proposed Co_3_O_4_/GCE were found to be close to 100%. The stability and reproducibility could be seen from the relative standard deviation (RSD) data as given in Table 1. All concrete values of RSD were less than 1%, suggesting the potential practical application of the as developed MGCE for the determination of ascorbic acid. It further confirms the real sample analysis of the as presented non-enzymatic ascorbic acid sensor. The RSD (%) was calculated as the (standard deviation/mean of measured data by 3 repeated measurements) ×100%.

For simplicity, we provided the comparison analysis of our presented results with the reported works as shown in Table 2. The quantitative information about the performance of the presented non-enzymatic ascorbic acid sensor was collected by the comparative analysis as given in Table 2 [4,13,14,15,16,17,18,19,20,21,22,23,23,24,25,26,27,28,29,30,31,32,33,34,35,36,37,38,39,40,41,42]. From the previous studies about the determination of ascorbic acid, it can be seen that the composite systems that were used are complicated and highly expensive to characterize compared to the presented non-enzymatic ascorbic acid sensor. The Co_3_O_4_ nanostructures (sample-3) demonstrated a wide linear range, which was verified by different electrochemical modes, confirming their high potential toward practical applications. This comparative analysis revealed a better performance of Co_3_O_4_ nanostructures (sample-3), which can be connected to the strong surface modification of Co_3_O_4_ nanostructures by the citrate anions. The improved performance of the Co_3_O_4_ nanostructures could be attributed to the surface defects and surface roughness as verified by SEM analysis. Moreover, the citrate ion treated Co_3_O_4_ nanostructures showed significant crystal quality, thereby playing a vital role in driving the ascorbic oxidation reaction.

## 4. Conclusions

In this study, we employed the low cost surface modifying agent of sodium citrate with a high density of oxygenated terminals and it significantly modified the surface properties of the Co_3_O_4_ nanostructures. Furthermore, the citrate ions enhanced the electrochemical properties of the Co_3_O_4_ nanostructures toward the development of a non-enzymatic ascorbic acid sensor. The structural analysis was carried out using low resolution SEM and powder XRD techniques. The treated Co_3_O_4_ nanostructures exhibited a rough surface compared to the pristine Co_3_O_4_ nanostructures, confirming the significant surface modifications. The Co_3_O_4_ nanostructures were observed to have enhanced crystal properties. The Co_3_O_4_ nanostructures have been proven to be an excellent protocol for the sensitive and selective determination of ascorbic acid due to their strong surface roughness, high surface area, and excellent compatibility with the surface of GCE. The linear range of the Co_3_O_4_ nanostructures for the ascorbic acid detection was found to be from 0.5 mM to 6.5 mM using the CV and LSV electrochemical modes, respectively. Furthermore, the Co_3_O_4_ nanostructures were characterized with a low limit of detection of 0.001 mM, high stability, and selectivity. The surface modification of Co_3_O_4_ nanostructures using oxygenated groups is a facile, low cost, and ecofriendly approach for a wide range of applications such as biomedical, energy conversion, and storage systems.

## Figures and Tables

**Table 1:** Recovery (%) of ascorbic acid from the urine sample using the proposed Co_3_O_4_/GCE non-enzymatic sensor.

Sample (Urine)	Added (mM)	Found (mM)	Recovery (%)	RSD (%)
1	0.523	0.521 ± 0.0085	100.38	0.471
2	1.084	1.091 ± 0.0068	100.64	0.563
3	1.532	1.541 ± 0.0075	100.58	0.524

**Table 2:** The comparative study of the presented ascorbic acid detection results with the already published results of ascorbic acid using different materials.

Electrode Material	Linear Range (µM)	Detection Limit (µM)	Reference
Pd/CNF-CPE ^a^	50–4000	15	[26]
Chitosan–graphene	50–1200	50	[34]
OMC/Nafion ^b^	40–800	20	[35]
Carbon nanotube voltametric	80–1360	20	[36]
Nitrogen doped graphene (NG)/GCE	5–1300	2.2	[37]
Au/Ru nanoshells/GCE ^c^	5–2000	2.2	[2]
RGO/GCE ^ d ^	30–350	14.8	[38]
Ferrocene methanol/CNTY	3–3000	1.32	[39]
MWCNT/CCE ^e^	15–800	7.71	[40]
RGO–ZnO/GCE ^f^	50–2350	3.71	[41]
RGO-CD-MWCNT-POM ^g^	5–2000	0.84	[42]
Co_3_O_4_/GCE	500–6500	1	This work

^a^ Palladium particles deposited on the carbon nanofiber and used for the modification of the carbon paste electrode. ^b^ Ordered mesoporous carbon/Nafion composite film. ^c ^Gold nanoparticles functionalized beta cyclodextrin graphene oxide onto the glass carbon electrode. ^d ^Reduced graphene oxide/glassy carbon electrode. ^e^ MWCNTs: carboxylated multi-walled carbon nanotubes, PANI: polyaniline. ^f ^Reduced graphene oxide-ZnO/glassy carbon electrode. ^g^ Reduced graphene oxide/*β*-cyclodextrin/multiwall carbon nanotubes/polyoxometalate.

## Data Availability

All research data is included in this article.
